# Yangyin Fuzheng Jiedu prescription as an adjunct to minimally invasive treatment in early-stage hepatocellular carcinoma: a randomized controlled trial

**DOI:** 10.3389/fphar.2026.1780139

**Published:** 2026-07-06

**Authors:** Huiwen Yan, Xinhui Wang, Xiaoli Liu, Lihua Yu, Yuqing Xie, Tong Wu, Yuyong Jiang, Zhiyun Yang

**Affiliations:** 1 Center for Integrative Medicine, Beijing Ditan Hospital, Capital Medical University, Beijing, China; 2 Department of Traditional Chinese Medicine, Beijing Children’s Hospital, Capital Medical University, Beijing, China; 3 Laboratory for Clinical Medicine, Capital Medical University, Beijing, China

**Keywords:** hepatocellular carcinoma, minimally invasive treatment, randomized controlled trial, recurrence-free survival, yangyin fuzheng jiedu prescription

## Abstract

**Background and Aims:**

Recurrence remains common after minimally invasive treatment in patients with early-stage hepatocellular carcinoma (HCC). This randomized controlled trial evaluated the efficacy and safety of Yangyin Fuzheng Jiedu Prescription (YFJP) as an adjunct to minimally invasive treatment in early-stage HCC.

**Method:**

In this randomized controlled trial, 300 patients with early-stage HCC (BCLC stage 0–A) undergoing minimally invasive treatment were randomly assigned (1:1) to receive YFJP plus minimally invasive treatment (YFJP group) or minimally invasive treatment alone (Control group). Patients were followed for 48 weeks. The primary endpoint was recurrence-free survival (RFS). RFS was assessed using the Kaplan-Meier method and compared with the log-rank test. Cox proportional hazards models were used for exploratory subgroup analyses. Safety was evaluated by the incidence of adverse events.

**Result:**

At 48 weeks, outcome data were available for 135 patients in the YFJP group and 137 patients in the Control group. Recurrence-free survival was significantly longer in the YFJP group than in the Control group (log-rank p = 0.016). Subgroup analyses showed a generally consistent direction of treatment effect across clinical subgroups, although the magnitude of effect varied. The incidence of adverse events was similar between groups, with no unexpected safety signals observed.

**Conclusion:**

In patients with early-stage HCC undergoing minimally invasive treatment, adjunctive treatment with YFJP was associated with improved recurrence-free survival over 48 weeks without compromising safety. These findings support the potential role of YFJP as an adjunctive therapeutic option in early-stage HCC.

**Clinical Trial Registration:**

https://clinicaltrials.gov/, Identifier NCT04264962.

## Introduction

Hepatocellular carcinoma (HCC) remains a leading cause of cancer-related mortality worldwide, representing a substantial global health burden (Bray et al., 2024; Singal et al., 2023). In Asia, the epidemiology of HCC is evolving, with changes in viral hepatitis control, population aging, and surveillance practices contributing to an increasing proportion of patients being diagnosed at an early stage (Han et al., 2024; Zhang et al., 2022; Qi et al., 2023). For these patients, curative-intent minimally invasive treatments such as local ablation and transarterial chemoembolization (TACE) are commonly applied. However, despite effective initial tumor control, recurrence after treatment remains frequent, reflecting the intrinsic biological aggressiveness of HCC and the pro-tumorigenic background of chronic liver disease and cirrhosis. Post-treatment recurrence therefore remains a major obstacle to durable disease control and long-term survival in patients with early-stage HCC.

Current post-treatment management strategies for early-stage HCC rely predominantly on surveillance, and there is a lack of established adjuvant therapies that effectively reduce recurrence risk while preserving liver function. Although systemic targeted therapies and immune-based treatments have improved outcomes in advanced HCC, their role in the adjuvant setting remains limited by safety concerns, cost, and uncertain benefit in patients with preserved liver function. In this context, adjunctive therapeutic approaches have attracted increasing interest. Notably, a multicentre randomized clinical trial demonstrated that adjuvant Huaier granule significantly reduced recurrence after curative resection of HCC(Chen et al., 2018). In addition, observational studies and systematic reviews have suggested that adjunctive traditional Chinese medicine may be associated with improved long-term outcomes in patients with HCC(Liu et al., 2019; Pu et al., 2022). Nevertheless, high-quality evidence from randomized controlled trials evaluating adjunctive interventions in early-stage disease remains limited, particularly in patients undergoing minimally invasive treatment.

Yangyin Fuzheng Jiedu Prescription (YFJP) is a compound herbal formulation that has been widely used in clinical practice as supportive therapy for patients with chronic liver disease and HCC. Experimental studies and network pharmacology analyses have suggested that YFJP may exert biological activity relevant to HCC, including immunomodulatory effects (Yan et al., 2020; 2021; Xie et al., 2023). However, despite its widespread clinical use, the efficacy of YFJP as an adjunct to minimally invasive treatment in early-stage HCC has not been evaluated in a randomized controlled design. To address this evidence gap, we conducted a randomized controlled trial to assess the efficacy and safety of Yangyin Fuzheng Jiedu Prescription as an adjunct to minimally invasive treatment in patients with early-stage HCC, with recurrence-free survival as the primary endpoint over a 48 week follow-up period.

## Patients and methods

### Study design and participants

We conducted a prospective randomized controlled trial (RCT) registered at ClinicalTrials.gov (registration number: NCT04264962), enrolling 300 patients with small hepatocellular carcinoma at Beijing Ditan Hospital, Capital Medical University, from July 2017 to June 2020. The patients were randomly allocated in a 1:1 ratio to receive either minimally invasive treatment alone or YFJP in addition to minimally invasive treatment. The random allocation sequence was generated using the PROC PLAN procedure in SAS software version 9.2, with a 1:1 allocation ratio. No stratification factors were used in the randomization procedure. Allocation concealment was implemented using sequentially numbered, opaque, sealed envelopes. After written informed consent had been obtained and eligibility had been confirmed, the next envelope in sequence was opened to determine group assignment. Because YFJP was administered as a botanical decoction with distinctive appearance, taste, and odor, double-blinding and placebo control were not feasible in this pragmatic randomized trial. To reduce detection bias, tumor recurrence was determined based on contrast-enhanced CT or MRI reports reviewed by clinicians who were not involved in treatment allocation and were unaware of treatment assignment. The imaging reports did not contain information on group allocation. The inclusion criteria included: (1) age eligibility (≤75 years old), (2) meeting the criteria for hepatocellular carcinoma, (3) specific size and multi-centricity of the main nodule (single lesion, three nodules ≤3 cm), (4) being post-transarterial chemoembolization (TACE) or radiofrequency ablation (RFA), (5) surgery cannot be allowed, (6) providing informed consent. The exclusion criteria involved (1) severe heart, lung, or kidney dysfunction, (2) pregnancy or breastfeeding, (3) mental or cognitive disorders, (4) participation in other drug trials, and (5) known allergy to the study drug. The diagnostic criteria for small HCC adhered to the Asia-Pacific clinical guidelines for small HCC(Omata et al., 2017). The study was conducted in compliance with local regulatory requirements, Good Clinical Practice, and the Declaration of Helsinki, and was approved by the Ethics Committee of Ditan Hospital, with written informed consent obtained from all participants. The key contents of the informed consent form are provided in Supplementary Material.

### Interventions and minimally invasive treatment

All patients received minimally invasive treatment for early-stage hepatocellular carcinoma, including radiofrequency ablation (RFA), transarterial chemoembolization (TACE), or combined TACE plus RFA. The choice of RFA, TACE, or combined TACE plus RFA was made by the treating physicians according to routine clinical decision-making based on tumor burden, tumor location, liver function, vascular anatomy, and technical feasibility. All minimally invasive procedures were performed at Beijing Ditan Hospital by experienced physicians according to institutional standard procedures.

Patients assigned to the YFJP group received YFJP orally at 150 mL per dose, twice daily, after minimally invasive treatment when clinically stable. YFJP was planned to continue for 48 weeks unless intolerable adverse events, withdrawal of consent, poor adherence, or other protocol-defined discontinuation occurred. Temporary interruption or discontinuation of YFJP was allowed when patients developed clinically significant adverse events, poor tolerance, or were unable to continue treatment. Treatment adherence was assessed during follow-up based on patient interviews, prescription or dispensing records, and medical records.

### Composition and quality control of YFJP

YFJP consisted of the following botanical drugs: Adenophora tetraphylla (Thunb.) Fisch. or Adenophora stricta Miq. [Campanulaceae; Adenophorae Radix, Nanshashen, 12 g], Ophiopogon japonicus (Thunb.) Ker Gawl. [Asparagaceae; Ophiopogonis Radix, Maidong, 12 g], Astragalus membranaceus (Fisch.) Bunge var. Mongholicus (Bunge) P.K. Hsiao [Fabaceae; Astragali Radix, Sheng Huangqi, 30 g], Atractylodes macrocephala Koidz. [Asteraceae; Atractylodis Macrocephalae Rhizoma, Baizhu, 12 g], Bupleurum chinense DC. [Apiaceae; Bupleuri Radix, Beichaihu, 9 g], Sophora flavescens Aiton [Fabaceae; Sophorae Flavescentis Radix, Kushen, 9 g], Cynanchum paniculatum (Bunge) Kitag. [Apocynaceae; Cynanchi Paniculati Radix et Rhizoma, Xuchangqing, 12 g], Paris polyphylla Sm. var. yunnanensis (Franch.) Hand.-Mazz. or Paris polyphylla Sm. var. chinensis (Franch.) H. Hara [Melanthiaceae; Paridis Rhizoma, Chonglou, 9 g], Akebia quinata (Thunb.) Decne., Akebia trifoliata (Thunb.) Koidz., or Akebia trifoliata (Thunb.) Koidz. var. australis (Diels) Rehder [Lardizabalaceae; Akebiae Fructus, Bayuezha/Yuzhizi, 15 g], Isodon rubescens (Hemsl.) H. Hara [Lamiaceae; Rabdosiae Rubescentis Herba, Donglingcao, 30 g], and Oldenlandia diffusa (Willd.) Roxb. [Rubiaceae; Baihua Sheshecao, dried whole herb, 30 g]. (Yan et al., 2021). All drug purchase and quality inspection are strictly carried out in accordance with the Pharmacy Management system of the Department of Pharmacy of Beijing Ditan Hospital. All the Chinese herbal medicine used in this study were decocted by the Pharmacy of Traditional Chinese Medicine of Beijing Ditan Hospital affiliated to Capital Medical University. The equipment, personnel, methods and management of the substitute decoction room are in line with the “Management Standard of traditional Chinese Medicine Room of Medical institutions” issued by the State Administration of traditional Chinese Medicine of the Ministry of Health.

To further evaluate the chemical quality and batch-to-batch consistency of YFJP, UHPLC-Q Exactive MS-based fingerprint analysis was performed on nine batches of YFJP decoction. The samples were analyzed in both positive and negative ion modes. Common peak matching and fingerprint similarity evaluation were performed using the Similarity Evaluation System for Chromatographic Fingerprint of Traditional Chinese Medicine. A reference fingerprint was generated using the average method with multipoint correction.

In the positive ion mode, 22 common peaks were detected, and four representative marker metabolites were identified by comparison with reference standards, retention times, accurate mass, and MS/MS fragmentation patterns, including matrine, calycosin-7-O-glucoside, oridonin, and paeonol. In the negative ion mode, 34 common peaks were detected, and five representative marker metabolites were identified, including deacetylasperulosidic acid methyl ester, calycosin-7-O-glucoside, Mutong phenylethanoid glycoside B, ononin, and saikosaponin B1.

Batch-to-batch consistency was assessed based on fingerprint similarity. The similarities between the nine batches and the reference fingerprint ranged from 0.973 to 0.999 in the positive ion mode and from 0.981 to 0.995 in the negative ion mode. All similarity values were greater than 0.95, indicating good batch-to-batch chemical consistency of YFJP. Detailed chromatographic conditions, common peak matching results, representative fingerprints, identified marker metabolites, and similarity values are provided in the Supplementary Apprndix S1.

### Procedures

Clinical and laboratory evaluations were conducted at baseline, 24 weeks and 48 weeks respectively, and adverse events were evaluated at 24 weeks and 48 weeks. Follow-up assessments included clinical evaluation, liver function tests, blood biochemical tests, serum alpha-fetoprotein measurement, and contrast-enhanced CT or MRI examination according to the study protocol and routine clinical practice. Tumor recurrence was determined by contrast-enhanced CT or MRI. Imaging-based recurrence was assessed based on typical radiological features of hepatocellular carcinoma, including arterial phase hyperenhancement and portal venous or delayed phase washout when applicable, with reference to mRECIST principles. Pathological confirmation was not routinely required unless imaging findings were inconclusive.

For patients who did not return for scheduled visits, telephone follow-up was attempted to determine survival status, recurrence-related information if available, and reasons for discontinuation or loss to follow-up.

### Outcomes

The primary endpoint was the recurrence-free survival (RFS) within 48 weeks after initial minimally invasive treatment. RFS was defined as the time from initial minimally invasive treatment to the first documented tumor recurrence. The date of starting treatment was defined as the zero time-point, the patients who were lost to follow-up were censored at the last date they were known to be alive; and the patients who remained alive were censored at 48 weeks after starting treatment. Adverse events (AEs) were collected during follow-up and summarized by event type and overall incidence. The relationship between adverse events and YFJP was evaluated according to their temporal relationship with YFJP administration, known adverse reactions, alternative explanations, improvement after discontinuation, and recurrence after re-exposure when applicable.

### Statistical analyses

The primary efficacy analysis was performed in the intention-to-treat population, which included all randomized patients according to their assigned treatment group, regardless of treatment adherence, protocol deviations, or follow-up completion. Patients who withdrew consent or were lost to follow-up during the 48 week follow-up were censored at the last available follow-up assessment in the time-to-event analyses. Safety analyses were performed in patients with available adverse-event assessments during follow-up.

All statistical analyses were performed using the R software, version 4.2.2 (http://www.r-project.org/). Continuous variables are expressed as medians (IQRs) and were compared using the Mann-Whitney U test. Categorical variables are presented as numbers (percentages) and were compared using the chi-square or Fisher’s exact test. No imputation was performed for the missing data. Recurrence-free survival and overall survival were estimated using the Kaplan-Meier method and compared by the log-rank test. Hazard ratios (HRs) and 95% CIs were estimated using a Cox proportional-hazard model. P value < 0.05 was considered statistically significant.

## Results

### Baseline characteristics of study participants

**FIGURE 1:**
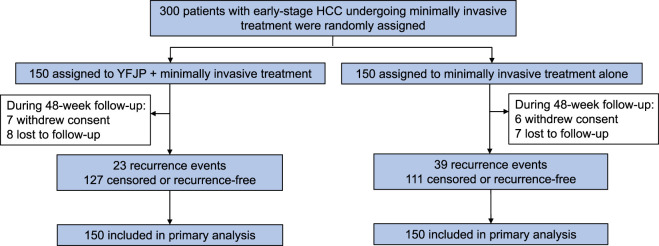
Study design and patient flow. All randomized patients were included in the primary analysis. Patients who withdrew consent or were lost to follow-up during the 48 week follow-up were censored at the last available follow-up assessment. Recurrence events were defined as the first documented tumor recurrence during follow-up. HCC, hepatocellular carcinoma; YFJP, Yangyin Fuzheng Jiedu Prescription.

A total of 300 patients with early-stage HCC undergoing minimally invasive treatment were randomized, with 150 assigned to the YFJP plus minimally invasive treatment group and 150 assigned to the minimally invasive treatment alone group. All randomized patients were included in the ITT population for the primary analysis. The attrition was balanced between groups, with 15 patients excluded from the YFJP group and 13 from the Control group. In the YFJP group, seven patients withdrew consent and eight were lost to follow-up; in the Control group, six patients withdrew consent and seven were lost to follow-up (Figure 1).

The baseline characteristics of the ITT population are summarized in Table 1. The mean age of the study population was 57.20 ± 9.31 years. Overall, 240 patients were male (80.0%) and 60 were female (20.0%), with an approximate male-to-female ratio of 4:1. In the overall ITT population, 23 patients received RFA, 77 received TACE, and 200 received combined TACE plus RFA. The distribution of minimally invasive treatment modalities was balanced between the YFJP and Control groups (P = 0.292). Baseline demographic characteristics, past medical history, complications, tumor-related characteristics, liver function-related variables, and minimally invasive treatment modalities were generally balanced between the two groups (all P ≥ 0.05). Laboratory characteristics of the ITT population after 24 weeks of treatment are shown in Supplementary Table S1.

**TABLE 1:** Baseline clinical characteristics of enrolled patients in the ITT population.

Characteristics	Category	Total (n = 300)	YFJP (n = 150)	Control (n = 150)	P Value
Age, years	​	56.83 ± 8.65	57.00 ± 8.28	56.66 ± 9.03	0.734
Gender, male	​	240 (80.0)	121 (80.7)	119 (79.3)	0.885
Smoking history	​	180 (60.0)	93 (62.0)	87 (58.0)	0.556
Hypertension	​	72 (24.0)	37 (24.7)	35 (23.3)	0.892
Coronary artery disease	​	12 (4.0)	7 (4.7)	5 (3.3)	0.768
Diabetes mellitus	​	84 (28.0)	45 (30.0)	39 (26.0)	0.520
Etiology	HBV	256 (85.3)	131 (87.3)	125 (83.3)	0.644
	HCV	17 (5.7)	7 (4.7)	10 (6.7)	​
	Alcohol abuse	18 (6.0)	9 (6.0)	9 (6.0)	​
	NAFLD	9 (3.0)	3 (2.0)	6 (4.0)	​
Child-pugh stage	A	251 (83.7)	127 (84.7)	124 (82.7)	0.755
	B	49 (16.3)	23 (15.3)	26 (17.3)	​
Type of treatment	RFA	23 (7.7)	15 (10.0)	8 (5.3)	0.292
	TACE	77 (25.7)	39 (26.0)	38 (25.3)	​
	TACE + RFA	200 (66.7)	96 (64.0)	104 (69.3)	​
Number of tumors	Single nodule	156 (52.0)	85 (56.7)	71 (47.3)	0.133
	Multiple nodules	144 (48.0)	65 (43.3)	79 (52.7)	​
BCLC stage	0	31 (10.3)	19 (12.7)	12 (8.0)	0.255
​	A	269 (89.7)	131 (87.3)	138 (92.0)	​
Laboratory data					
White blood cell count (10^9^/L)	​	4.10 (2.80, 5.60)	4.10 (2.85, 5.40)	4.13 (2.76, 5.62)	0.985
Hemoglobin (g/L)	​	129.00 (108.97, 144.00)	130.50 (110.50, 145.75)	126.50 (107.50, 140.38)	0.081
Platelet (10^9^/L)	​	84.75 (53.00, 134.25)	85.50 (56.25, 145.85)	84.75 (51.08, 119.62)	0.294
Creatinine (µmol/L)	​	67.15 (56.27, 79.23)	68.10 (58.85, 79.20)	65.90 (55.10, 79.22)	0.239
ALT (U/L)	​	25.85 (17.90, 39.10)	26.05 (17.45, 38.90)	25.85 (18.35, 38.98)	0.833
AST (U/L)	​	30.85 (22.80, 49.12)	30.45 (22.30, 48.95)	33.05 (23.75, 49.42)	0.451
Total Bilirubin (µmol/L)	​	18.90 (13.00, 29.18)	17.60 (12.83, 27.30)	19.10 (13.03, 31.08)	0.205
Albumin (g/L)	​	38.40 (35.30, 42.00)	38.90 (35.73, 41.60)	37.70 (34.85, 42.18)	0.173
GGT (U/L)	​	55.30 (27.82, 89.95)	52.70 (25.65, 87.35)	61.25 (29.53, 98.00)	0.250
Cholinesterase (U/L)	​	4480.50 (2796.25, 6493.00)	4677.50 (2997.75, 6617.25)	4137.50 (2608.75, 6400.00)	0.241
PTA(%)	​	81.00 (68.75, 92.25)	81.00 (68.25, 92.75)	81.00 (70.00, 91.75)	0.604
INR	​	1.17 (1.06, 1.31)	1.16 (1.06, 1.31)	1.17 (1.06, 1.31)	0.852
AFP (ng/mL)	​	10.30 (2.90, 152.57)	7.95 (2.73, 378.82)	11.85 (3.30, 111.85)	0.993

Continuous variables are presented as mean ± SD, or median (IQR), as appropriate. Categorical variables are presented as n (%). Between-group comparisons were performed using the Student’s t-test for normally distributed continuous variables, the Mann–Whitney U test for non-normally distributed continuous variables, and the chi-square test or Fisher’s exact test for categorical variables, as appropriate. A two-sided P value < 0.05 was considered statistically significant.

Abbreviations: AFP, alpha-fetoprotein; ALT, alanine aminotransferase; AST, aspartate aminotransferase; BCLC, barcelona clinic liver cancer; GGT, γ-glutamyl-transferase; HBV, hepatitis B virus; HCV, hepatitis C virus; INR, international normalized ratio; PTA, prothrombin time activity; TACE, transarterial chemoembolization.

### Recurrence-free survival in the overall population

**FIGURE 2:**
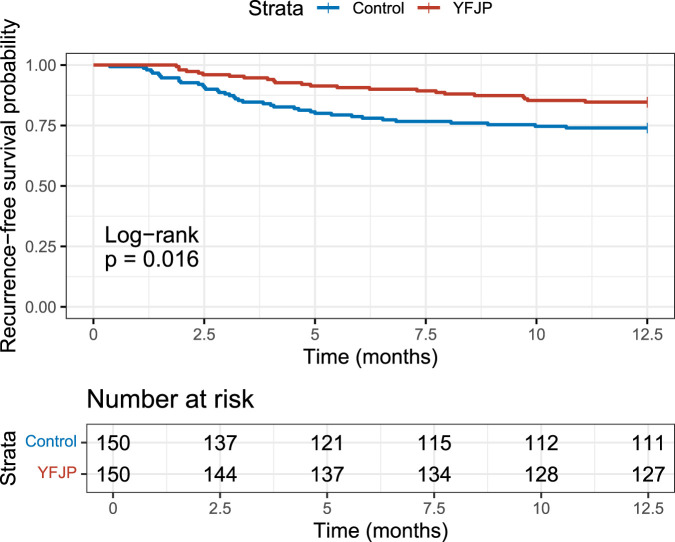
Recurrence-free survival among all randomized patients. Kaplan–Meier curves show recurrence-free survival over 48 weeks in patients treated with Yangyin Fuzheng Jiedu Prescription (YFJP group) versus standard treatment alone (Control group). Between-group differences were assessed using the log-rank test. Numbers at risk are shown below the plot.

During the 48 week follow-up, RFS was significantly longer in the YFJP group than in the Control group. The estimated RFS rates at week 24 were 90.7% (95% CI, 86.1–95.4) in the YFJP group and 78.7% (95% CI, 72.4–85.5) in the Control group. By week 48, RFS remained higher in the YFJP group than in the Control group (84.7% [95% CI, 79.1–90.6] vs. 74.0% [95% CI, 67.3–81.4]). The median RFS was not reached in either group during the 48 week follow-up. Kaplan-Meier analysis showed a significant between-group difference in RFS (log-rank P = 0.016; Figure 2). Cox proportional hazards analysis showed that YFJP treatment was associated with a significantly lower risk of recurrence than control treatment (HR, 0.54; 95% CI, 0.32–0.90; P = 0.018).

### Subgroup analyses of recurrence-free survival

**FIGURE 3:**
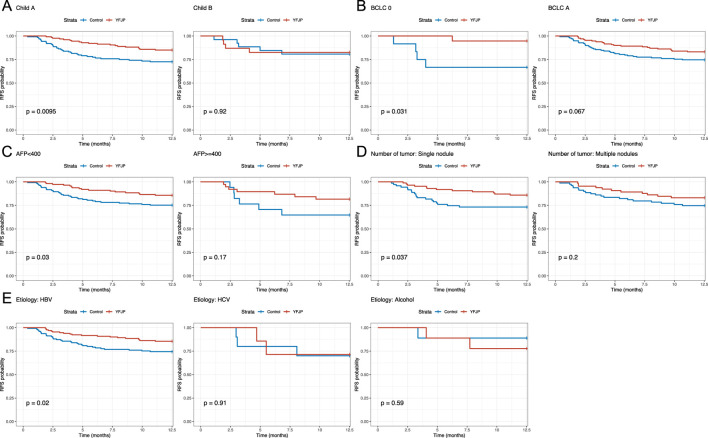
Subgroup analyses of recurrence-free survival (RFS). Kaplan–Meier curves depict RFS over 48 weeks in patients receiving Yangyin Fuzheng Jiedu Prescription (YFJP group) versus standard treatment alone (Control group). Subgroup analyses were performed according to **(A)** Child–Pugh class **(A,B)**, **(B)** BCLC stage (0, **(A)**, **(C)** serum alpha-fetoprotein level (AFP <400 vs. ≥400 ng/mL), **(D)** tumor number (single vs. multiple nodules), and **(E)** etiology (HBV, HCV, alcohol-related). Between-group differences within each subgroup were assessed using the log-rank test, with P values shown in each panel. RFS was defined as the time from initial treatment to tumor recurrence or last follow-up.

Prespecified subgroup analyses were performed to explore the consistency of the treatment effect. Kaplan-Meier analyses showed a numerically favorable RFS for the YFJP group in several subgroups, including patients with Child–Pugh class A, BCLC stage 0, AFP <400 ng/mL, HBV-related disease, and single tumors. In contrast, no statistically significant differences in RFS were observed in patients with Child–Pugh class B, BCLC stage A, AFP ≥400 ng/mL, or non-HBV etiologies. Log-rank p values for each subgroup comparison are shown in Figure 3. To explore the potential influence of HBV replication status on recurrence, subgroup analyses were performed according to baseline HBV DNA status. The direction of treatment effect remained consistent in both HBV DNA-positive and HBV DNA-negative patients, although no statistically significant differences were observed within either subgroup (Supplementary Figure S2).

**FIGURE 4:**
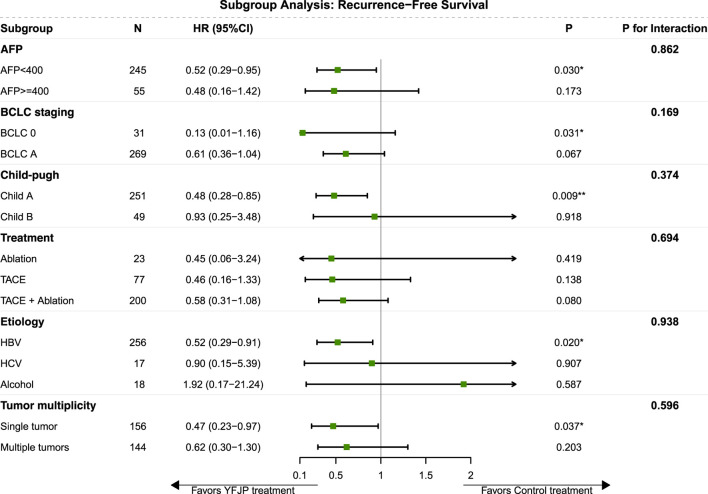
Forest plot of recurrence-free survival (RFS). Hazard ratios (HRs) and 95% confidence intervals (CIs) for RFS comparing the YFJP group with the Control group across predefined subgroups are shown. HRs were estimated using Cox proportional hazards models. The vertical dashed line indicates an HR of 1.0, with values < 1.0 favoring the YFJP group. Subgroup analyses were exploratory, and no adjustment for multiple comparisons was performed.

Cox proportional hazards regression analyses further evaluated the consistency of treatment effects across predefined subgroups. Forest plot analyses demonstrated that hazard ratios for RFS generally favored the YFJP group, although the magnitude of effect varied and several confidence intervals crossed unity (Figure 4). No significant interactions between treatment effect and subgroup variables were observed.

### Safety

Adverse events occurred at similar frequencies in the YFJP group and the Control group. No statistically significant differences were observed between groups for overall adverse events or for individual adverse event categories (Table 2). There were no unexpected safety signals associated with the addition of YFJP during the 48 week follow-up period.

**TABLE 2:** Adverse events and laboratory abnormalities during the 48 week follow-up.

Adverse events	YFJP (n = 150)		Control (n = 150)		P value
	Grade 1–2	Grade ≥3	Grade 1–2	Grade ≥3	
Adverse event	28 (18.7)	0	22 (14.7)	0	0.353
Fatigue	2 (1.3)	0	1 (0.7)	0	0.562
Fever	5 (3.3)	0	5 (3.3)	0	1
Diarrhea	6 (4.0)	0	4 (2.7)	0	0.52
Nausea	4 (2.7)	0	4 (2.7)	0	1
Dyspepsia	5 (3.3)	0	3 (2.0)	0	0.474
^†^Abnormal liver function	6 (4.0)	0	5 (3.3)	0	0.759

Adverse events are presented as n (%). Between-group comparisons were performed using the chi-square test or Fisher’s exact test, as appropriate. Adverse events were graded according to the Common Terminology Criteria for Adverse Events (CTCAE). No Grade 3 or higher adverse events were observed in either group during the 48 week follow-up period. A two-sided P value < 0.05 was considered statistically significant.

^†^ One or more than one of the liver function panel results were abnormal. The liver function panel includes alanine aminotransferase, aspartate aminotransferase, total bilirubin, direct bilirubin, albumin, globulin, serum creatinine, etc.

## Discussion

In this randomized controlled trial, adjunctive YFJP improved 48 week recurrence-free survival and reduced the risk of recurrence in patients with small HCC undergoing minimally invasive treatment, without compromising safety.

Despite advances in surveillance and locoregional treatment, recurrence after curative-intent treatment remains a major challenge in HCC, particularly in patients with chronic liver disease and cirrhosis (Bosch et al., 2004; Rahbari et al., 2011). In the present trial, adjunctive YFJP was associated with a higher estimated 48 week RFS rate than control treatment (84.7% vs. 74.0%) and a significantly lower risk of recurrence (HR, 0.54; 95% CI, 0.32–0.90). These findings are consistent with our previous experimental study showing that YFJP alleviated T cell exhaustion and immunosuppression in tumor-bearing mice, as well as our retrospective clinical study suggesting that nourishing-yin, strengthening-vital-qi, and detoxifying Chinese patent medicine may reduce macrovascular invasion after TACE (Yan et al., 2021; Yan et al., 2024). Compared with previous observational studies and systematic reviews of adjunctive TCM in HCC, the present randomized trial provides more direct clinical evidence that YFJP may improve early post-treatment recurrence control after minimally invasive therapy (Wang et al., 2023; Pu et al., 2022).

Mechanistically, the clinical findings of this trial suggest that the benefit of YFJP may involve both tumor-related and immune microenvironment-related effects. The improved RFS and reduced recurrence risk observed in the YFJP group indicate a potential effect on early post-treatment recurrence, which may be influenced by residual tumor activity, intrahepatic inflammation, and the cirrhotic background liver. This interpretation is supported by previous experimental evidence showing that several botanical drugs and representative metabolites in YFJP exert antitumor or immunoregulatory effects. Sophora flavescens Aiton has been reported to have antitumor properties, and Kushen injection can act on macrophages and CD8+T cells to reshape the immune microenvironment of HCC, thereby improving the therapeutic effect of low-dose sorafenib and reducing chemotherapy-related adverse reactions (Yang et al., 2020). In addition, systematic analysis also showed that Hedyotis diffusa Wild. can achieve anti-tumor effects by improving the immune microenvironment (Han et al., 2020). These findings are also consistent with the view that combining TCM with standard HCC treatments may represent a promising strategy for reducing recurrence and improving RFS(Luo et al., 2021). However, because immune cell subsets, cytokine profiles, and tumor microenvironmental markers were not prospectively measured in the present trial, this immunomodulatory explanation should be interpreted as a biologically plausible hypothesis rather than direct mechanistic proof.

In addition to RFS, YFJP was associated with favorable changes in liver function-related parameters, including increased albumin and decreased total bilirubin levels. These changes are clinically relevant because most patients in this trial had cirrhosis, and preservation of hepatic functional reserve may contribute to better post-treatment recovery and recurrence control. Previous studies have shown that Astragalus polysaccharides can increase albumin levels by enhancing immune function and protecting liver cells (Ibrahim et al., 2022) and Wuling powder containing Atractylis macrocephala (Koidz.) Hand.-Mazz. can increase albumin in patients with ascites (Mou et al., 2022). Similar improvements in liver function parameters have also been reported in other studies on TCM treatment (Zhao et al., 2014; Liao et al., 2020). In addition, Bupleurum chinense DC. has been reported to regulate inflammation, immunity, and metabolism in chronic liver diseases (Zhong et al., 2024), and Dachaihu Tang can alleviate cholestasis and reduce bilirubin levels (Xu et al., 2022). Together with the observed decrease in AFP in the YFJP group, these laboratory changes suggest that YFJP may improve the post-treatment liver condition while reducing tumor-related activity. AFP is widely used in HCC surveillance, and its decrease may be consistent with improved disease control after minimally invasive treatment. This interpretation is supported by previous systematic reviews on the effect of TCM on tumor markers in HCC (Shi et al., 2017; She et al., 2021), as well as experimental evidence suggesting that Hedyotis diffusa Willd. may inhibit tumor cell growth and promote apoptosis (Chen et al., 2021).

Several limitations should be acknowledged. First, although this study showed improvements in RFS, AFP, albumin, and total bilirubin, immune cell subsets, cytokine profiles, and tumor microenvironmental markers were not prospectively assessed. Therefore, the biological mechanisms underlying the observed clinical benefit of YFJP could not be directly confirmed. Second, the 48 week follow-up period mainly captured early post-interventional recurrence and was insufficient to evaluate long-term oncological outcomes, including overall survival. Third, the single-center design may limit the generalizability of our findings, and multicenter validation in more diverse patient populations is needed. Fourth, double-blinding and placebo control were not feasible because YFJP was administered as a botanical decoction with distinctive appearance, taste, and odor. This open-label design may have introduced performance bias, although detection bias was reduced by objective imaging-based recurrence assessment conducted by radiologists blinded to treatment allocation.

## Conclusion

In this randomized controlled trial, adjunctive treatment with Yangyin Fuzheng Jiedu Prescription in patients with early-stage hepatocellular carcinoma undergoing minimally invasive therapy was associated with improved recurrence-free survival over a 48 week follow-up period, without an increase in adverse events. These findings suggest that YFJP may represent a feasible and well-tolerated adjunctive option in the post-treatment management of early-stage HCC.

## Data Availability

The raw data supporting the conclusions of this article will be made available by the authors, without undue reservation.
