# Supplementary material for: Yangyin Fuzheng Jiedu prescription as an adjunct to minimally invasive treatment in early-stage hepatocellular carcinoma: a randomized controlled trial
Source: Front Pharmacol. 2026 Jul 6;17:1780139. doi: 10.3389/fphar.2026.1780139 (PMC13381619; doi:10.3389/fphar.2026.1780139)
Supplement: Supplementary file 1 [file Table1.docx]

Table S1. Laboratory characteristics of the ITT population after 24 weeks of treatment.

| Characteristics | Total(n=300) | YFJP (n=150) | Control (n=150) | P value |
| --- | --- | --- | --- | --- |
| White blood cells (10^9^/L) | 4.26 (2.84, 5.92) | 4.38 (2.86, 6.16) | 4.18 (2.79, 5.58) | 0.437 |
| Hemoglobin (g/L) | 131.55 (109.12, 145.00) | 135.00 (113.00, 149.25) | 128.00 (104.25, 143.95) | 0.136 |
| Platelets (10^9^/L) | 87.80 (55.00, 134.52) | 87.80 (54.75, 137.95) | 88.00 (55.25, 129.00) | 0.853 |
| Creatinine (µmol/L) | 67.15 (58.08, 82.47) | 67.25 (59.05, 79.85) | 66.65 (56.37, 83.25) | 0.71 |
| ALT (U/L) | 23.75 (17.20, 36.95) | 23.40 (16.58, 35.68) | 25.45 (18.02, 41.17) | 0.23 |
| AST (U/L) | 31.30 (23.55, 46.08) | 29.65 (22.08, 41.10) | 32.70 (24.05, 53.00) | 0.05 |
| Total Bilirubin (µmol/L) | 20.00 (13.00, 34.92) | 18.80 (12.30, 30.22) | 22.35 (13.95, 42.98) | 0.011 |
| Albumin (g/L) | 38.45 (32.08, 42.80) | 38.75 (33.68, 42.82) | 37.00 (30.68, 42.68) | 0.06 |
| GGT (U/L) | 40.55 (23.82, 74.60) | 40.30 (21.80, 70.25) | 41.45 (27.22, 81.83) | 0.181 |
| Cholinesterase (U/L) | 4932.00 (3009.00, 6560.25) | 5143.00 (3569.75, 6712.00) | 4628.50 (2465.50, 6485.50) | 0.097 |
| PTA(%) | 83.00 (70.25, 92.00) | 84.00 (73.00, 93.25) | 82.00 (65.25, 90.00) | 0.137 |
| INR | 1.15 (1.07, 1.30) | 1.14 (1.05, 1.27) | 1.15 (1.09, 1.35) | 0.088 |
| Alpha-fetoprotein(ng/ml) | 4.80 (2.40, 65.90) | 3.75 (2.08, 14.38) | 8.70 (2.70, 270.45) | 0.001 |

Continuous variables are presented as median (IQR). Between-group comparisons were performed using the Mann–Whitney U test. A two-sided P value <0.05 was considered statistically significant.

Abbreviations: AFP, alpha-fetoprotein; ALT, alanine aminotransferase; AST, aspartate aminotransferase; GGT, γ-glutamyl-transferase; INR, international normalized ratio; PTA, prothrombin time activity.
