# Supplementary material for: Yangyin Fuzheng Jiedu prescription as an adjunct to minimally invasive treatment in early-stage hepatocellular carcinoma: a randomized controlled trial
Source: Front Pharmacol. 2026 Jul 6;17:1780139. doi: 10.3389/fphar.2026.1780139 (PMC13381619; doi:10.3389/fphar.2026.1780139)
Supplement: Supplementary file 2 [file Supplementaryfile1.pdf]

Supplementary Appendix S1. Quality Control and Chemical Characterization of Yangyin Fuzheng Jiedu Prescription (YFJP)

1. Botanical Drug Information

Composition: Adenophora Radix, Ophiopogonis Radix, Astragali Radix, Atractylodis Macrocephalae Rhizoma, Bupleuri Radix, Sophorae Flavescentis Radix, Cynanchi Paniculati Radix et Rhizoma, Paridis Rhizoma, Akebiae Fructus, Rabdosiae Rubescentis Herba, and Hedyotis diffusa.

2. Drugs and Experimental Methods

2.1 Drugs

Reference standards of saikosaponin B1, acteoside B, calycosin-7-O-glucoside, atractylenolide III, paeonol, deacetylasperulosidic acid methyl ester, ononin, polyphyllin VIII, oridonin, and matrine were purchased from Chengdu Refuns Biotech Co., Ltd. (Chengdu, China). The purity of all reference standards was >98% as determined by HPLC.

The reference standards included saikosaponin B1 (catalog no. C-085-10mg, batch no. RFS-02308008), acteoside B (catalog no. M-051-10mg, batch no. RFS-02604013), calycosin-7-O-glucoside (catalog no. M-020-10mg, batch no. RFS-01902019), atractylenolide III (catalog no. B-035-10mg, batch no. RFS-02306029), paeonol (catalog no. D-002-10mg, batch no. RFS-11812016), deacetylasperulosidic acid methyl ester (catalog no. Q-097-10mg, batch no. RFS-11810018), ononin (catalog no. M-013-10mg, batch no. RFS-01904002), polyphyllin VIII (catalog no. C-039-10mg, batch no. RFS-02101015), oridonin (catalog no. D-031-10mg, batch no. RFS-11712015), and matrine (catalog no. K-002-10mg, batch no. RFS-11812016).

The botanical drugs, including Adenophorae Radix (Nanshashen), Ophiopogonis Radix (Maidong), Astragali Radix (Sheng Huangqi), Atractylodis Macrocephalae Rhizoma (Baizhu), Bupleuri Radix (Chaihu), Sophorae Flavescentis Radix (Kushen), Cynanchi Paniculati Radix et Rhizoma (Xuchangqing), Paridis Rhizoma (Chonglou), Akebiae Fructus (Bayuezha), Rabdosiae Rubescentis Herba (Donglingcao), and Hedyotis diffusa (Baihua Shecao) were supplied by the Pharmacy Department of Beijing Ditan Hospital, Capital Medical University.

2.2Experimental Methods

Chromatographic conditions

A Vanquish Flex UHPLC chromatograph (Thermo Fisher Scientific, Waltham, MA, USA) equipped with an ACQUITY UPLC HSS T3 column (2.1 mm (inner diameter) ×100 mm (length), 1.7 μm (particle dimension)) (Waters Corp., Milford, MA, USA) was used for separation. The mobile phase was consisted of water (0.1% formic acid, phase A) and acetonitrile (phase B) with a flow rate of 0.3 mL/min and the column temperature was 40°C. The elution gradient was shown in Table 3 and the injection volume was 6.0 μL.

Table 1 Elution gradient

Time (min)	Mobile phase	
	A (v%)	B (v%)
0	98	2
1.0	98	2
14.0	70	30
25.0	0	100
28.0	0	100
28.1	98	2
29.5	98	2

MS conditions

The MS data was collected by a hybrid quadrupole orbitrap mass spectrometer (Q Exactive, Thermo Fisher Scientific, Waltham, MA, USA) equipped with a HESI-II spray probe. The parameters were set as follows: positive ion source voltage 3.7 kV and negative ion source voltage 3.5 kV, heated capillary temperature 320°C, sheath gas pressure 30 psi, auxiliary gas pressure 10 psi, desolvation temperature 300°C. Both the sheath gas and the auxiliary gas were nitrogen. The collision gas was also nitrogen with a pressure of 1.5 mTorr. The data was acquired in “Full scan/dd-MS^2” mode. The

parameters of the full scan were set as follows: resolution 70000, auto gain control target 1×10^6, maximum isolation time 50 ms and m/z scan range 100 – 1500. The dd-MS^2 data was collected with the parameters of resolution 17500, auto gain control target 1×10^5, maximum isolation time 50 ms, top n (n≤10) most intense parent ions selected for fragmentation coupled with dynamic exclusion mechanism, isolation window of m/z 2, collision energy 10 V, 30 V, 60 V and intensity threshold 1×10^5.

3. Results

3.1 Establishment and Similarity Evaluation of Fingerprints

Nine batches of the botanical drug decoction were prepared according to the procedure described in Section 2.3.1 and analyzed under the chromatographic and mass spectrometric conditions described in Sections 2.3.2 and 2.3.3. Raw data files were exported as TXT files using Xcalibur 2.2 software. Full-spectrum peak matching was performed using the Similarity Evaluation System for Chromatographic Fingerprints of Traditional Chinese Medicine (Version 2012, China). A reference fingerprint chromatogram was generated using the multipoint correction mode and the mean chromatogram method with a retention-time window of 0.1 min.

The fingerprint chromatograms obtained in positive-ion mode are shown in Figure 1, in which 22 common peaks were identified. The fingerprint chromatograms obtained in negative-ion mode are shown in Figure 2, in which 34 common peaks were identified.

In the positive-ion mode, the similarities between samples S1–S9 and the reference fingerprint chromatogram were 0.981, 0.991, 0.999, 0.997, 0.973, 0.997, 0.997, 0.973, and 0.994, respectively. In the negative-ion mode, the corresponding similarities were 0.981, 0.989, 0.995, 0.992, 0.989, 0.995, 0.988, 0.989, and 0.995, respectively.

All nine batches exhibited similarity values greater than 0.95 in both positive- and negative-ion modes, indicating excellent batch-to-batch consistency of the botanical drug formulation and stable quality of the raw botanical materials.

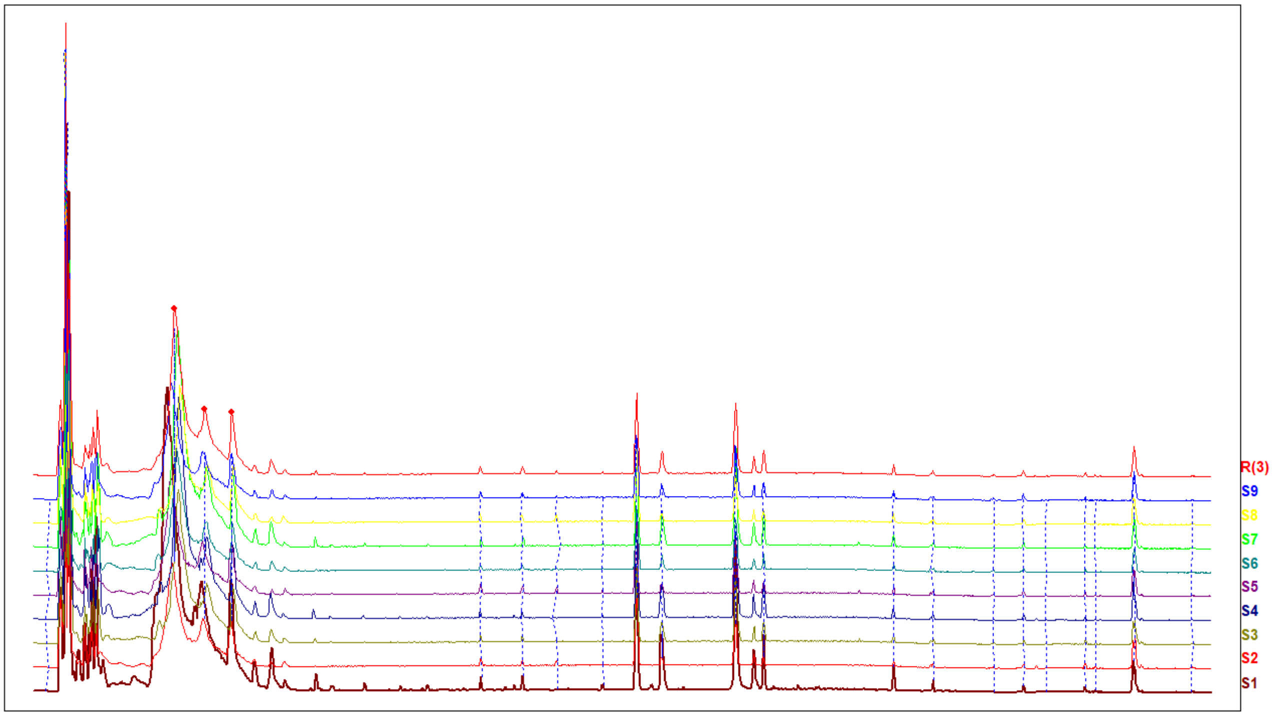

Figure 1. Fingerprint chromatograms of nine batches of Yangyin Fuzheng Jiedu Prescription (YFJP) decoctions in positive-ion mode.

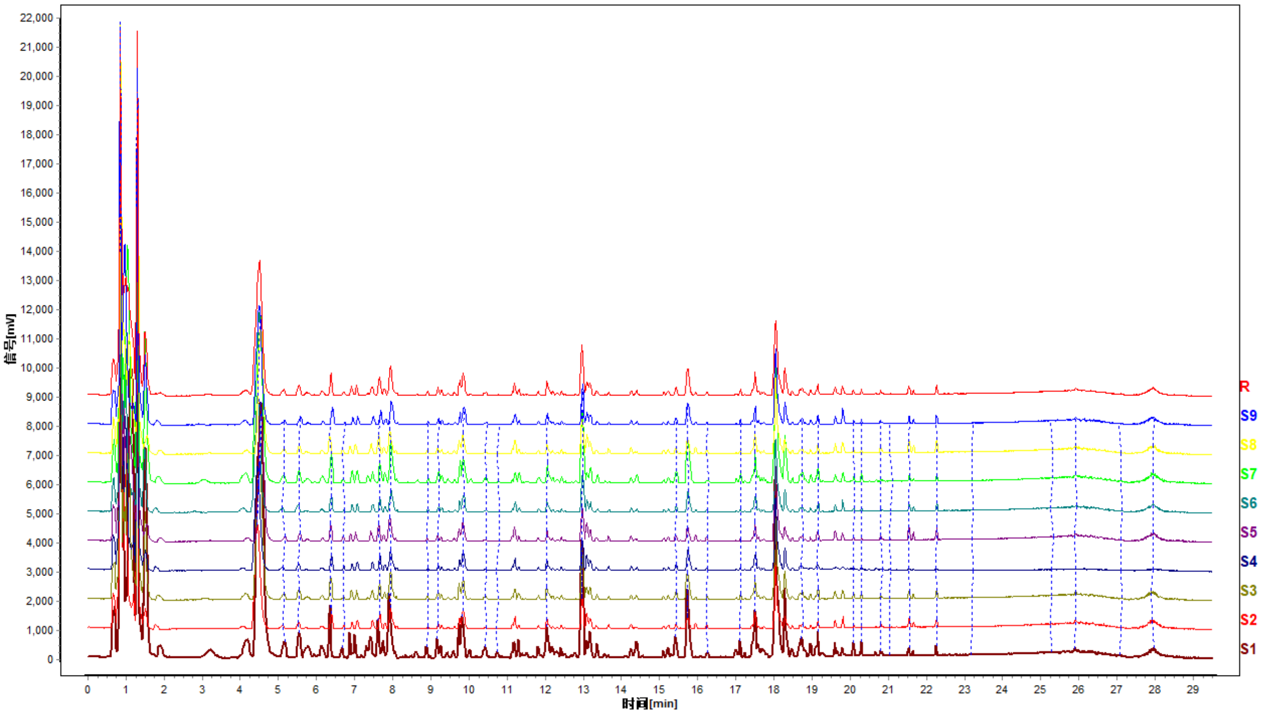

Figure 2. Fingerprint chromatograms of nine batches of Yangyin Fuzheng Jiedu Prescription (YFJP) decoctions in negative-ion mode.

3.2 Identification of Common Peaks in the Fingerprint Chromatograms

Based on the chromatographic retention times of the compounds in the mixed reference standard solution, together with high-resolution MS and MS/MS data, a total of four common peaks in the positive-ion mode and five common peaks in the negative-ion mode were identified. The identification results are summarized in Tables 5 and 6.

In the positive-ion mode, the common peaks were identified by comparison with authentic reference standards as matrine, calycosin-7-O-glucoside, oridonin, and paeonol. In the negative-ion mode, the common peaks were identified as deacetylasperulosidic acid methyl ester, calycosin-7-O-glucoside, acteoside B, ononin, and saikosaponin B1.

The chromatograms of the mixed reference standard solution are shown in Figure 3, and the chromatograms of the botanical drug samples are shown in Figure 4. In both figures, the upper and lower panels represent the base peak chromatograms (BPCs) acquired in positive-ion mode and negative-ion mode, respectively.

Table 2. Identification of Common Peaks in the Fingerprint Chromatogram under Positive-Ion Mode

Peak No.	tR (min)	Observed m/z	Theoretical m/z	Error (ppm)	Molecular Formula	MS/MS Fragment Ions	Identified Compound
1	3.52	249.1964	249.1961	1.20	$C_{15}H_{24}N_2O$	249.1963,148.1123	Matrine
3	11.22	447.1294	447.1286	1.79	$C_{22}H_{22}O_{10}$	285.0759,270.0524,447.1292, 253.0497,137.0236	Calycosin-7-O-glucoside
6	15.13	365.1964	365.1959	1.37	$C_{20}H_{28}O_6$	347.1858,133.0650,301.1801, 329.1751	Oridonin
7	17.61	167.0706	167.0703	1.80	$C_9H_{10}O_3$	167.0705,149.0600,121.0650	Paeonol

Table 3. Identification of Common Peaks in the Fingerprint Chromatogram under Negative-Ion Mode

Peak No.	tR (min)	Observed m/z	Theoretical m/z	Error (ppm)	Molecular Formula	MS/MS Fragment Ions	Identified Compound
2	5.56	449.1305	449.1301	0.89	$C_{17}H_{24}O_{11}$	241.0715,449.1304,403.1258, 101.0219	Deacetylasperulosidic Acid Methyl Ester
3	11.22	491.1200	491.1195	1.02	$C_{22}H_{22}O_{10}$	283.0616,268.0381,491.1209, 239.0351	Calycosin-7-O-glucoside
4	11.85	477.140	477.140	0.42	$C_{23}H_{26}O_{11}$	161.0227,477.1418,269.0456,	Acteoside

		4	2			133.0275,431.0987	B
5	14.27	475.124<br>9	475.124<br>6	0.63	$C_{22}H_{22}O_9$	267.0665,252.0426,161.0226,<br>475.1248	Ononin
8	19.55	825.466<br>3	825.464<br>2	2.54	$C_{42}H_{68}O_{13}$	779.4610,617.4070,825.4657,71.0<br>113	Saikosapon<br>in B1

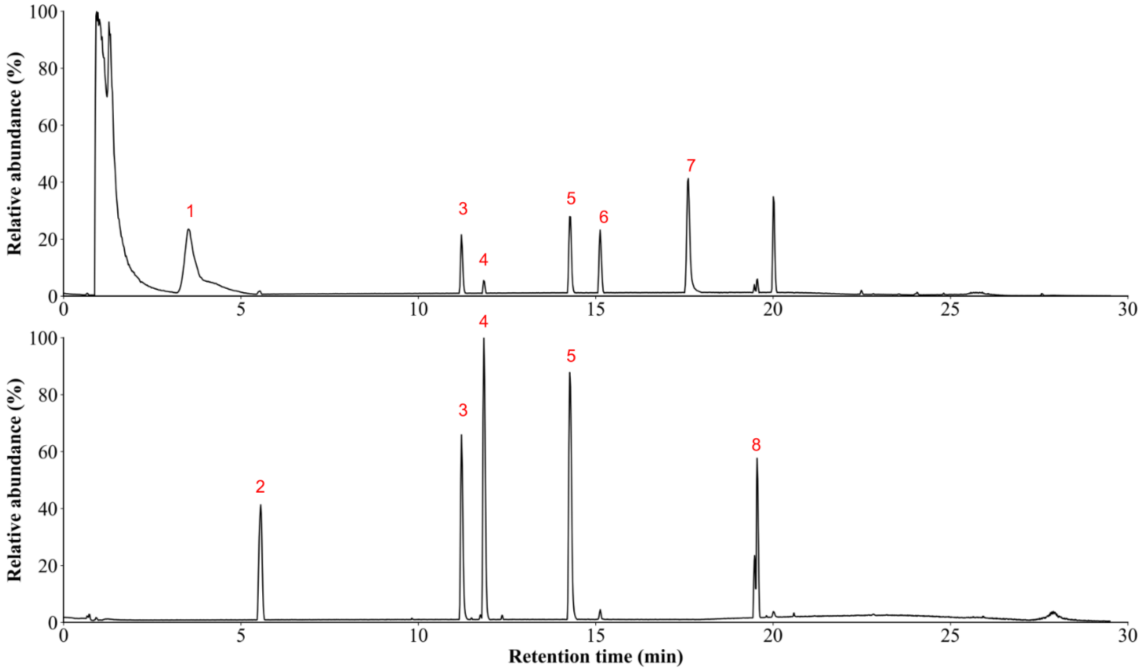

Figure 3. Base peak chromatograms (BPCs) of the mixed reference standard solution acquired in positive-ion mode (upper panel) and negative-ion mode (lower panel). Peak assignments: (1) Matrine; (2) Deacetylasperulosidic Acid Methyl Ester; (3) Calycosin-7-O-glucoside; (4) Acteoside B; (5) Ononin; (6) Oridonin; (7) Paeonol; and (8) Saikosaponin B1.

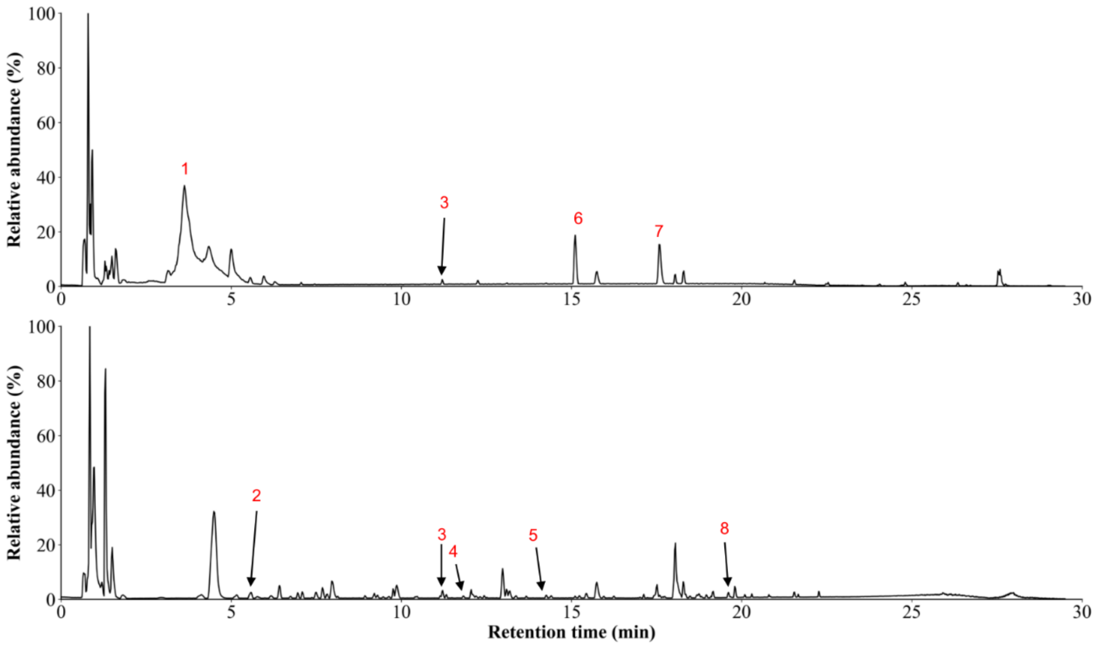

Figure 4. Base peak chromatograms (BPCs) of the Yangyin Fuzheng Jiedu Prescription (YFJP) sample acquired in positive-ion mode (upper panel) and negative-ion mode (lower panel). Peak assignments: (1) Matrine; (2) Deacetylasperulosidic Acid Methyl Ester; (3) Calycosin-7-O-glucoside; (4) Acteoside B; (5) Ononin; (6) Oridonin; (7) Paeonol; and (8) Saikosaponin B1.
