# Supplementary material for: Yangyin Fuzheng Jiedu prescription as an adjunct to minimally invasive treatment in early-stage hepatocellular carcinoma: a randomized controlled trial
Source: Front Pharmacol. 2026 Jul 6;17:1780139. doi: 10.3389/fphar.2026.1780139 (PMC13381619; doi:10.3389/fphar.2026.1780139)
Supplement: Supplementary file 3 [file Supplementaryfile2.docx]

Supplementary Figure. Subgroup analysis of recurrence-free survival according to baseline HBV DNA status. Kaplan–Meier curves comparing recurrence-free survival between the YFJP group and the Control group among patients with positive HBV DNA (left panel) and negative HBV DNA (right panel) at baseline. Between-group differences were assessed using the log-rank test. P values are shown in each panel.
